# Supplementary figures and images for: Evaluating the specificity of flavivirus proteases in Aedes aegypti cells for dengue virus 2-derived cleavage sites
Source: PLoS One. 2024 Dec 3;19(12):e0309095. doi: 10.1371/journal.pone.0309095 (PMC11614287; doi:10.1371/journal.pone.0309095)

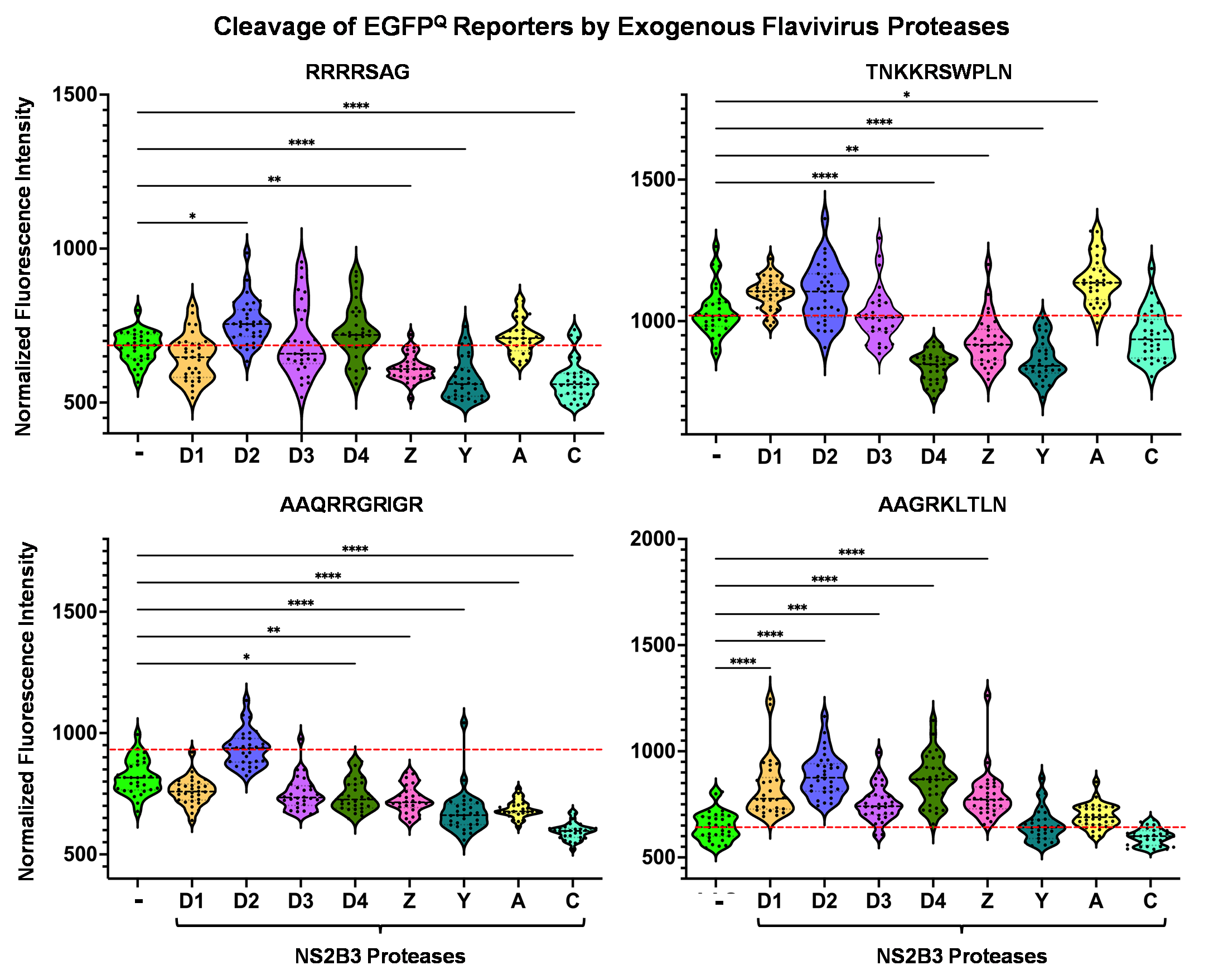

Supplement: S1 Fig — Cells were imaged 96 hours post-transfection at 40x magnification (n = 30). The results are the average of three experiments (*: ≤ 0.05, **: ≤ 0.01, ***: ≤ 0.001, ****: P ≤ 0.0001). Differences amongst the groups were analyzed statistically using a Kruskal-Wallis test, red horizontal line indicates the mean of the no-protease control sample. D1, DENV1; D2, DENV-2; D3, DENV-3; D4, DENV-4; Z, ZIKV; Y, YFV; A, AEFV; C, CFAV. (TIF) [file pone.0309095.s001.tif]

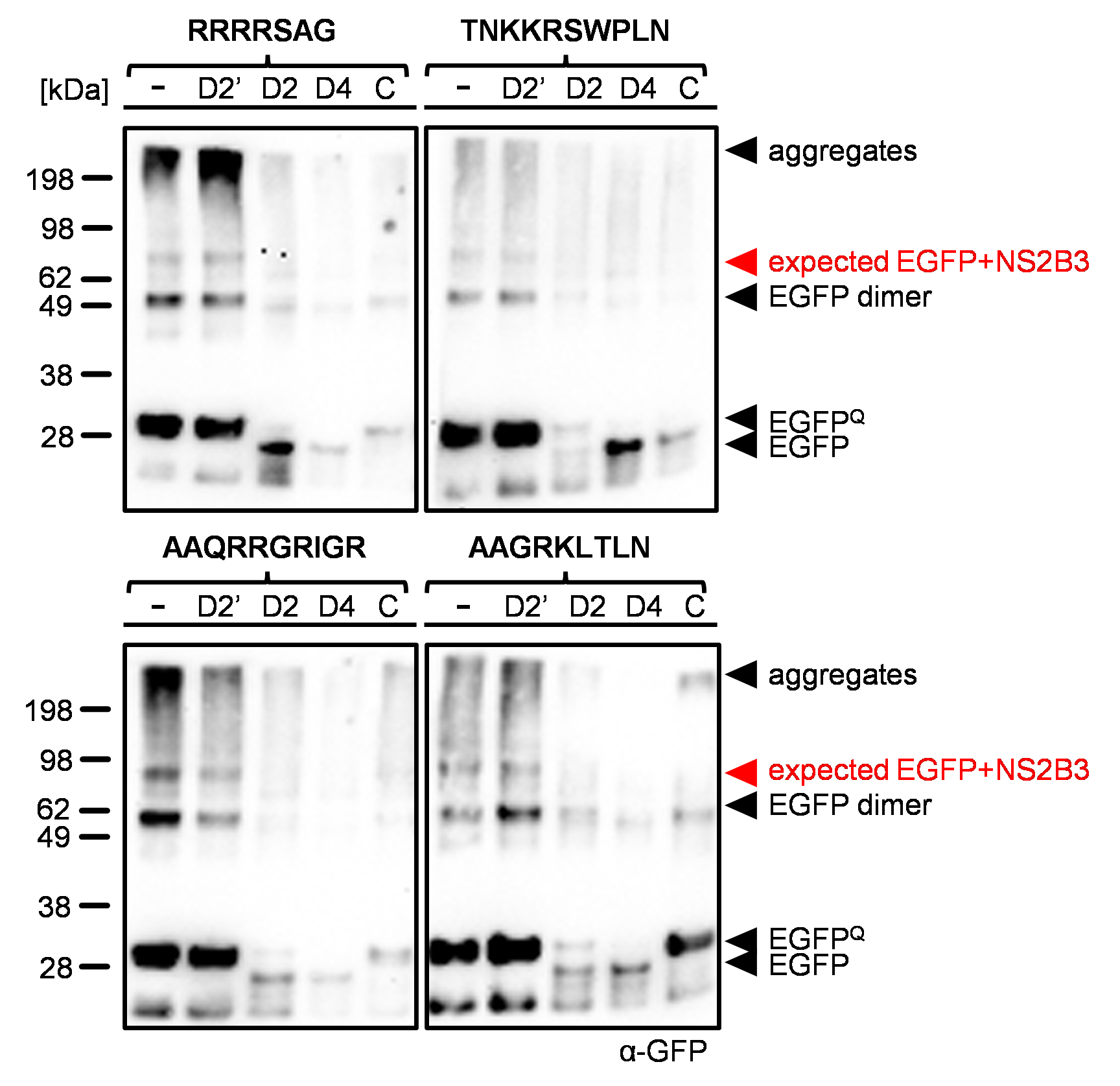

Supplement: S2 Fig — Western analysis of cell lysates following crosslinking (see Methods). Cells were transfected with the indicated reporter alone (-), or with the indicated protease (D2’, inactive DENV2 NS2B3; D2, DENV2; D4, DENV4; C, CFAV). Images were derived from different exposures. (TIF) [file pone.0309095.s002.tif]

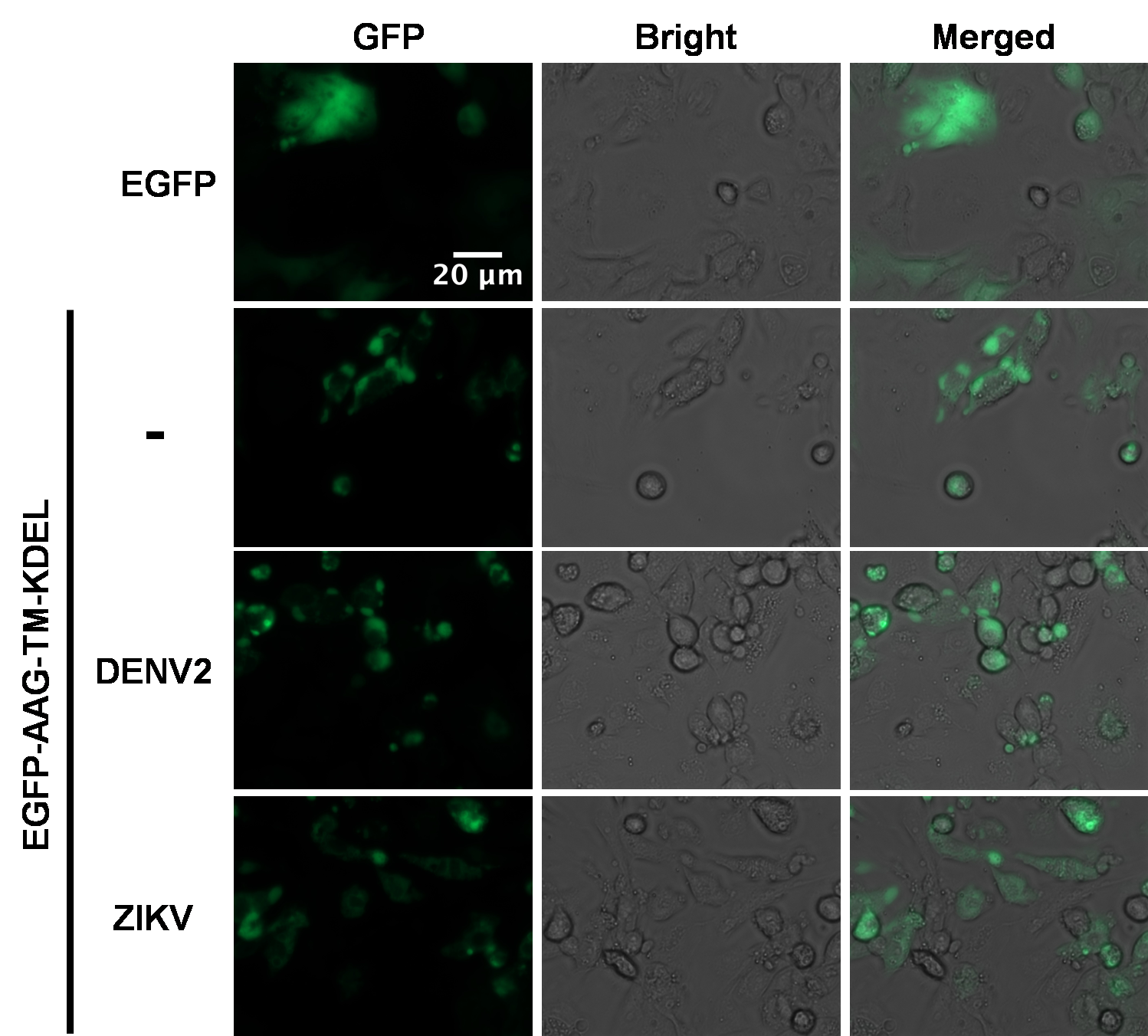

Supplement: S3 Fig — Virus-infected A20 cells expressing EGFP or EGFPTM. Cells were infected at an MOI of 0.01 with DENV2 or ZIKV 24 hours post-transfection and imaged 6 days post-infection at 40x magnification. (TIF) [file pone.0309095.s003.tif]

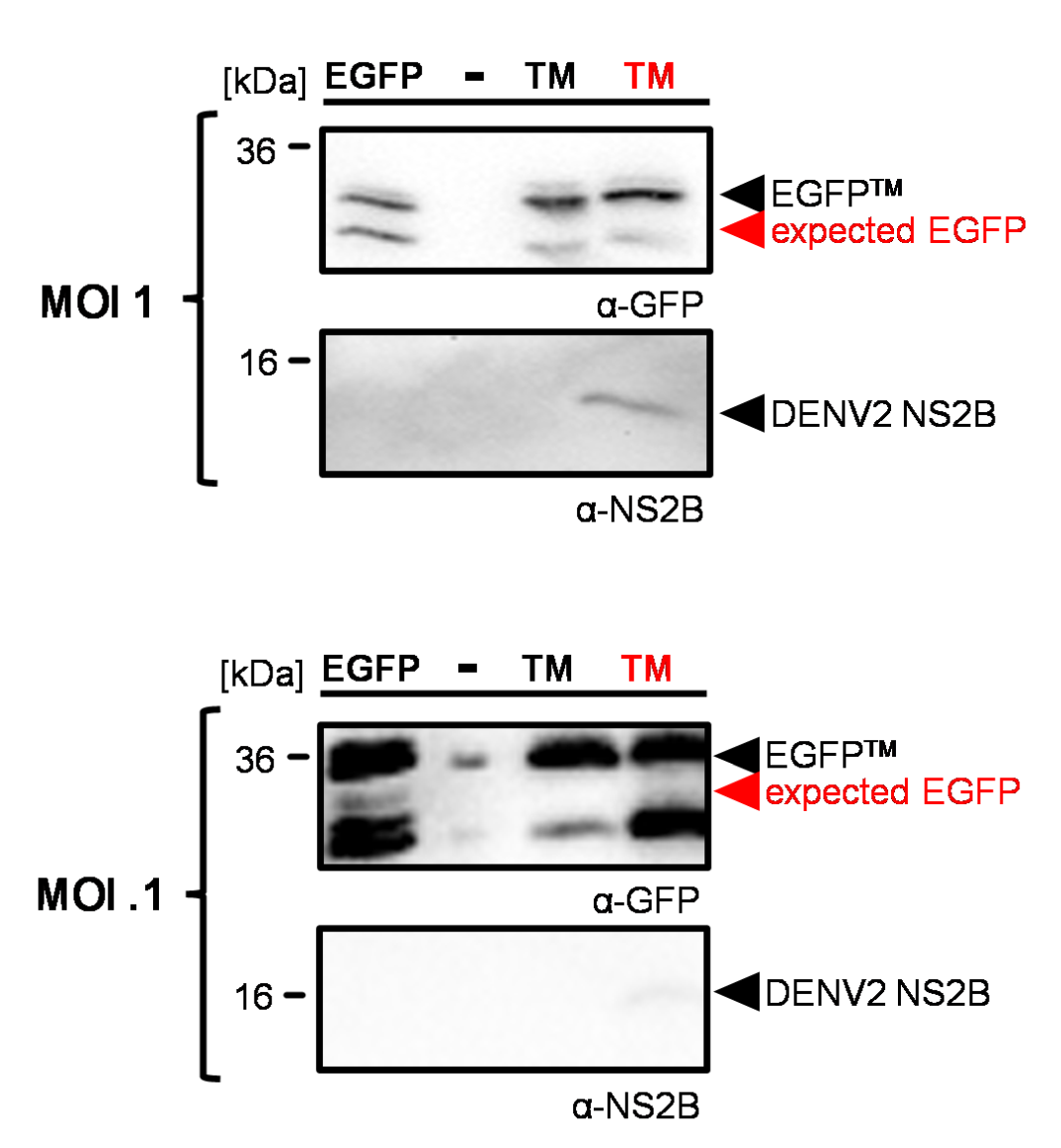

Supplement: S4 Fig — Virus-infected A20 cells expressing EGFP or EGFPTM. Cells were infected with DENV2 at an MOI of 0.1 and 1 24 hours post-transfection. Images were derived from different exposures. (TIF) [file pone.0309095.s004.tif]

Fig3a

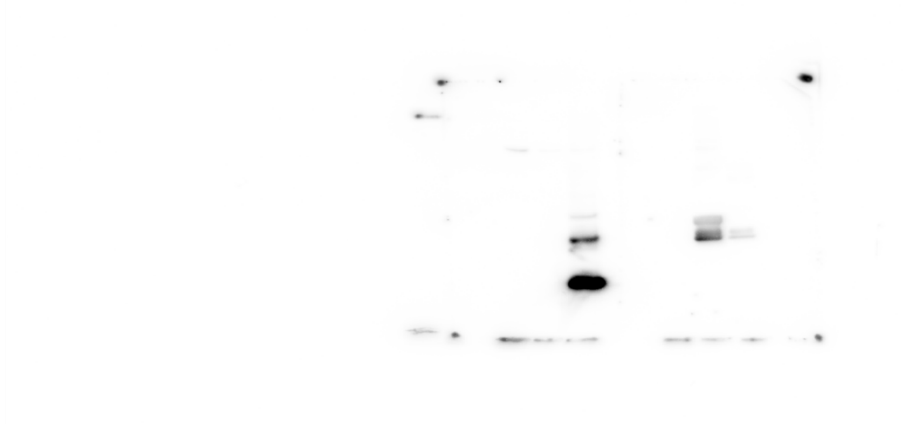

Fig3b

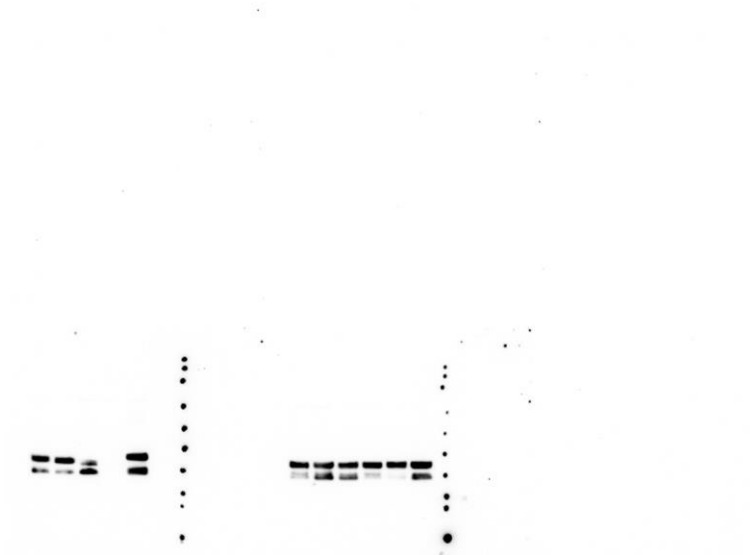

Fig3d

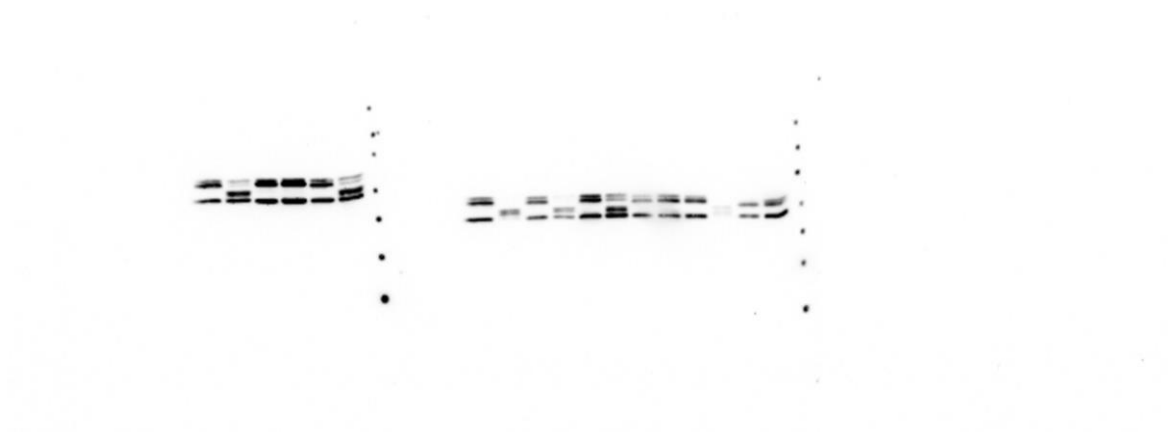

Fig3e

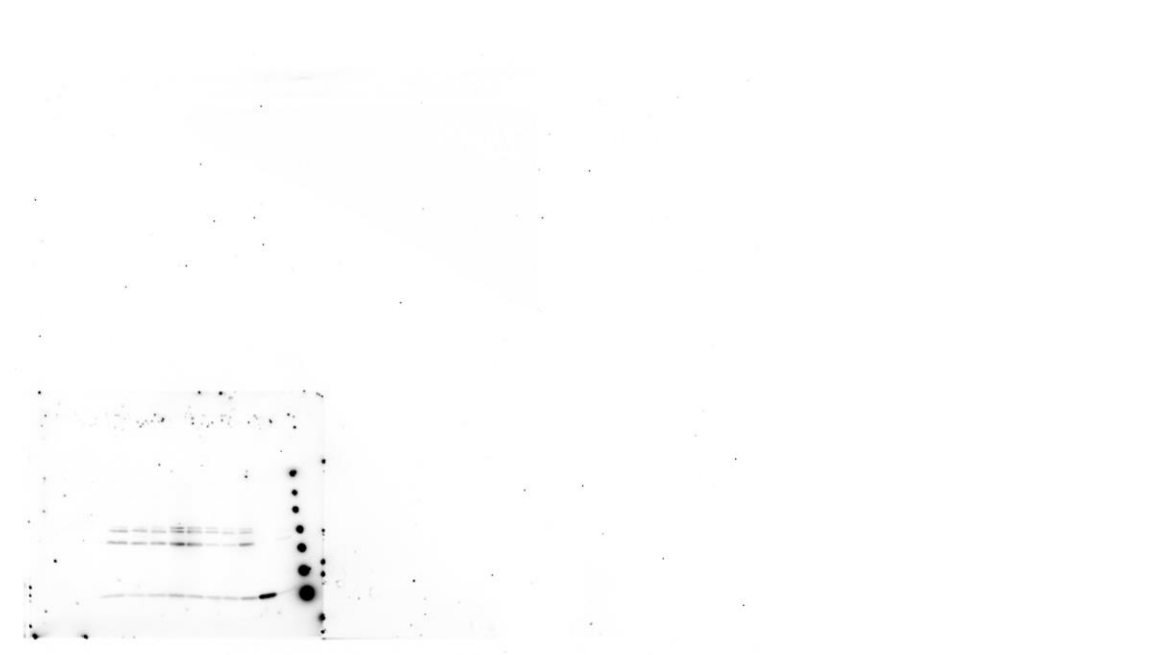

Fig4AAQ

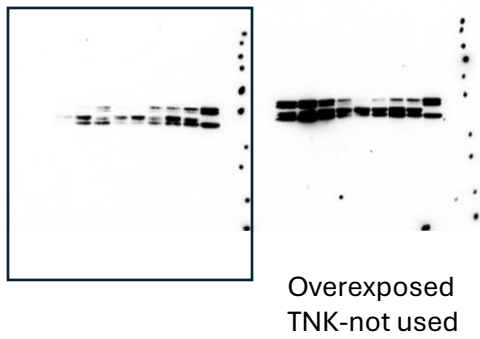

Fig4\_RRRSAG and AAG

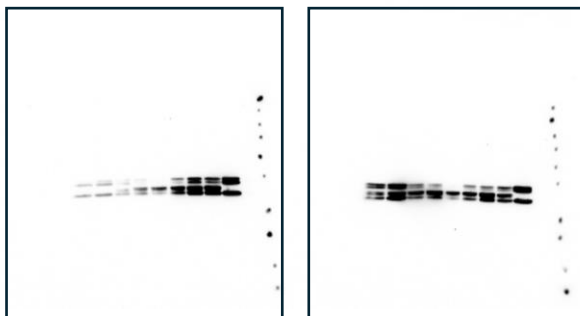

Fig4\_TNK

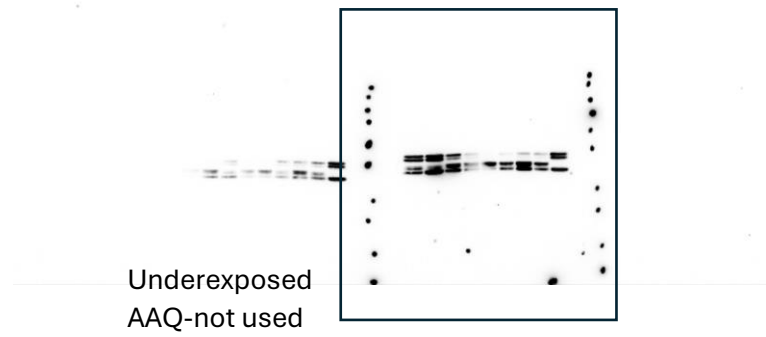

Fig5

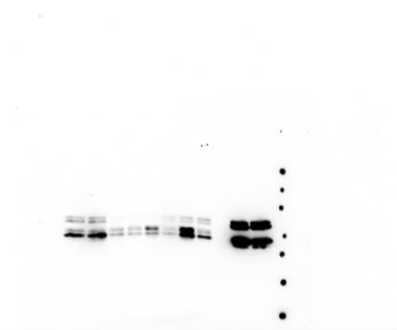

Fig6B\_EGFPB

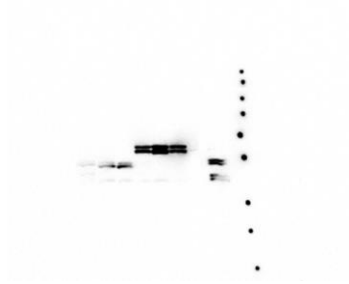

Fig6B\_EGFPTM

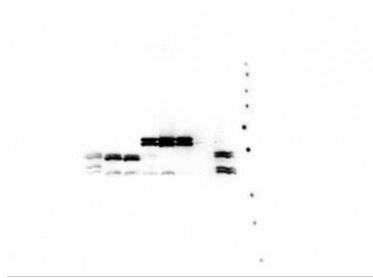

Fig6C

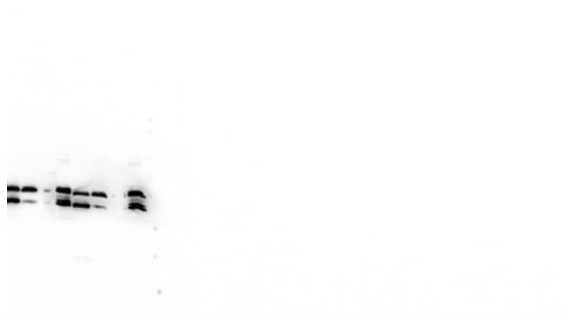

Fig6D

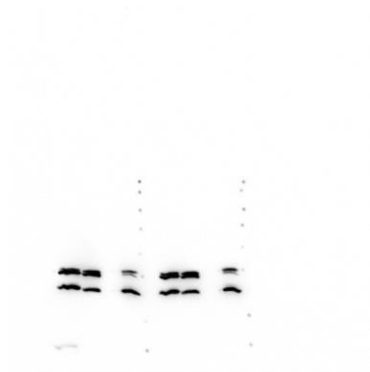

Fig\_S2

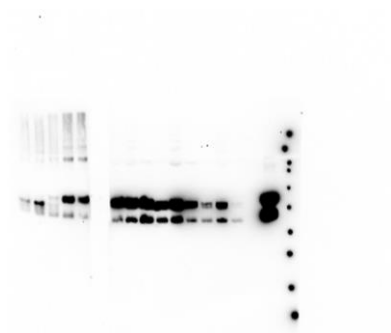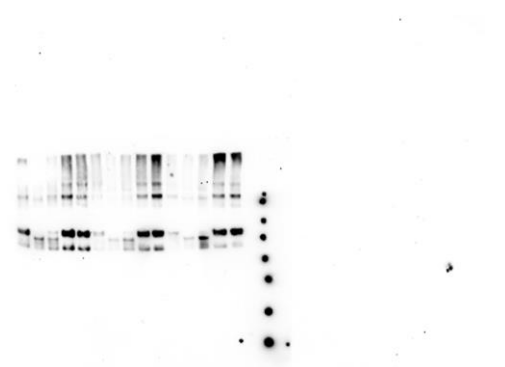

Supplement: S5 Fig — Raw, uncropped images of all Western blots used in this manuscript. (PDF) [file pone.0309095.s005.pdf]
