# Supplementary material for: Evaluating the specificity of flavivirus proteases in Aedes aegypti cells for dengue virus 2-derived cleavage sites
Source: PLoS One. 2024 Dec 3;19(12):e0309095. doi: 10.1371/journal.pone.0309095 (PMC11614287; doi:10.1371/journal.pone.0309095)
Supplement: Attachment — Submitted filename: Response_to_reviews.docx [file pone.0309095.s006.docx]

**Reviewer #1:** It is not clear the statement that “although reporters remained uncleaved during viral infection, this study provides foundation for further studies aimed at developing mosquitoes that are unable to transmit most, if not all flaviviruses”

*Response: This section was deleted and rephrased, as requested by the reviewer.*

The failed EGFPQ should be further explored to explain

*Response: It is not clear to what this is referring to. Maybe it is related to the point below, in which case, see corresponding response.*

Authors mention “When imaging, we did not observe the same sensitivity and consistency for reporting cleavage with any of the reporters when co-expressed with the exogenous proteases….” The corelation between immunofluorescence and western blot must be further explore and discuss

*Response: The reviewer does not provide a reason related to the technical conduct of our experiments as to why “the correlation between immunofluorescence and western blot must be further explored”. The EGFP-QP reporter system was developed and published by others. We adapted it to test additional cleavage sites and proteases. Our finding that in some cases this reporter system is not a reliable indicator of cleavage suggests a note of caution about relying solely on fluorescence values. Because it was unreliable, we backed up all of our experiments with Western blot data, which is less ambigious (proteins are either cleaved or not, while cleaved EGFP can be fluorescent or not depending on 3D conformation and fluorophore activation). It was not the focus of this manuscript to identify why this EGFP-QP reporter developed by others was not reliable for all constructs, nor do we make any conclusions in this regard. It seems like this is a question of IMPACT, where the reviewer feels the paper will be more IMPACTFUL if we solve this mystery, compared to if we just point out that the mystery exists. However, IMPACT is not a consideration at PLOS ONE.*

Quality of images need to be improved

*Response: High-quality images were included with the submission. The images appear to be low-quality due to compression by the PLoS One submission system. In order to view the high-quality versions, reviewers need to individually download each figure rather than viewing them in the PDF.*

Although the system seems a very interesting tool it must be further explored, and new controls must be introduced to give a better explanation on the observed phenotypes and make it a robust system

*Response: The main conclusions of this manuscript do not relate to the creation of a tool, despite that being part of our initial rationale. We present evidence that DENV2-based cleavage sites can be processed by a diverse array of flavivirus proteases when expressed outside of the context of an active infection. We also present evidence suggesting that such cleavage does not efficiently occur during an active infection. Our conclusions are based entirely on this evidence. The creation of a tool (ie, a system whereby toxic proteins can be produced specifically upon virus infection) will have to wait for this mystery to be resolved. Ultimately, this is a question of IMPACT. The reviewer appears to argue that developing a tool that works is more IMPACTFUL than failing to develop a tool while learning about flavivirus biology. This is irrelevant to the review criteria at PLOS ONE, where IMPACT is not a consideration. The remainder of the reviewer’s comment here cannot be productively addressed, due to a lack of information provided by the reviewer. The reviewer states that “new controls must be introduced”, but does not indicate what controls, for which experiments, and why such controls are needed beyond the controls we already included.*

Which is the difference between the 28 and the 38 KDa regarding functionality?

*Response: It is not clear to what this comment is referring to. In the case of our anti-EGFP antibody, we consistently observed two different sized fragments in all samples, and we do not currently understand why. This is not a subject of investigation of this manuscript. Importantly, all blots include a negative control, so we can easily discern those fragments from new fragments that appear when treating with viral protease.*

Figure 4 it is a central result and must be better explained

*Response: We have added additional information to the text here, though it would have been helpful had the reviewer indicated what specifically they found deficient in our initial explanation so our revisions could be more focused.*

**Reviewer #2:** In this study, the authors developed a cell-based reporter in Ae. aegypti A20 cells, adapting a technique originally used for monitoring flavivirus infection kinetics in mammalian cells. This approach was employed to assess the specificity of various flavivirus proteases to DENV2 cleavage sites in Ae. aegypti A20 cells. They demonstrated that proteases from six medically significant flaviviruses, as well as two more distantly related insect-specific flaviviruses, were able to cleave three sites derived from the DENV2 polyprotein, along with an additional consensus site.

Their cytoplasmic EGFPQ reporters, however, were not cleaved by the protease during viral infection with DENV2, likely due to virus-induced vesicle packets formed during infection. It is crucial to further investigate the underlying cause of this observation.

*Response: We agree, and this is an active area of investigation. However, this comment does not appear to be related to the technical conduct of our experiments, nor whether our conclusions are supported by our data, but rather is a statement on IMPACT. That is, it appears the reviewer feels the work will be of higher impact once this mystery is solved. While we agree, IMPACT is not a criterion for publication in PLoS One.*

The study also revealed that flavivirus NS3 proteases have the ability to process cleavage sites not native to their own genome. The authors suggest that their work contributes to the development of genetic control mechanisms aimed at preventing flavivirus transmission by insect vectors like Ae. aegypti. I recommend that the authors further discuss how this study could contribute to the development of such genetic control mechanisms.

*Response: We have provided further clarification of this in the Conclusions section, as suggested by the reviewer.*

Finally, I suggest increasing the resolution of the figures, as the quality of the fluorescence images is currently suboptimal.

*Response: High-quality images were included with the submission. The images appear to be low-quality due to compression by the PLoS One submission system. In order to view the high-quality versions, reviewers need to individually download each figure rather than viewing them in the PDF.*
