# Supplementary material for: Evaluating the specificity of flavivirus proteases in Aedes aegypti cells for dengue virus 2-derived cleavage sites
Source: PLoS One. 2024 Dec 3;19(12):e0309095. doi: 10.1371/journal.pone.0309095 (PMC11614287; doi:10.1371/journal.pone.0309095)
Supplement: Attachment — Submitted filename: Review typos in the abstract.docx [file pone.0309095.s007.docx]

- Review typos in the abstract “virusThese” “trans..”
- Would you consider to include in your discussion have frequent these proteolytic sites are in mosquito y mammalian cells proteins.
