# Supplementary material for: Evaluating the specificity of flavivirus proteases in Aedes aegypti cells for dengue virus 2-derived cleavage sites
Source: PLoS One. 2024 Dec 3;19(12):e0309095. doi: 10.1371/journal.pone.0309095 (PMC11614287; doi:10.1371/journal.pone.0309095)
Supplement: Attachment — Submitted filename: response2reviewers.docx [file pone.0309095.s008.docx]

Reviewer #1: -

Review typos in the abstract “virusThese” “trans..”

*Response: Corrected.*

- Since you are attempting to use it in transgenic refractory mosquitoes would you consider to include in your discussion have frequent these proteolytic sites are in mosquito cells proteins.

*Response: Our data indicate that in the absence of viral protease, there is no cleavage of the reporter by cellular proteins. It is possible that viral protease can cleave mosquito proteins, but this would already be occurring in a natural infection, and is not something we are attempting to control in these experiments.*

- For future work, how different promoters or enhancers would function for proteases activity

*Response: The relevance of the reviewer’s comment here is not clear. Additional promoters/enhancers to increase protease expression are not needed, as we readily observed cleavage of reporters with our current constructs. Lack of activity was only associated with virus infection, but in that case the amount of protease is solely dependent on viral replication, which is not controlled by promoter/enhancers.*

- If these proteases did not have effect on natural infections, which will be the way to go arount that

*Response: We added the following to the discussion “Competition between the reporter and the viral replication machinery may strongly favor the latter, leaving insufficient NS2B3 to cleave our reporter. It may be possible to make a more competetive reporter by using TM domains and protein components from the viral genome itself, rather than from ER-localized cellular proteins.”*

Reviewer #2:

Given that, in certain instances, this reporter system did not reliably indicate cleavage events, the authors should consider revising the title, potentially omitting the phrase 'using quenched-EGFP reporters.'

*Response: Title has been revised as recommended.*

This is my final recommendation for the manuscript. Moreover, it is worth noting that this reporter system has been previously published.

*Response: This is presented in the Introduction, lines 104-109, as well as lines 121-122.*

*“A cell-based fluorescent reporter was able to detect and monitor NS2B3 activity during flavivirus infection in live cells [38]. Flavivirus protease activity was detected by the resultant GFP fluorescence increase in cells that expressed fluorescently-quenched NS2B3 cleavable reporters [38]. Another study developed a dual-fluorescent reporter system to monitor flavivirus infection and endoplasmic reticulum (ER) manipulation [39].”*

*“This was achieved by adapting the previously described fluorescent activatable reporter of NS2B3 protease activity to assays in Ae. aegypti cells [38].”*
